# Deep Learning Powered Identification of Differentiated Early Mesoderm Cells from Pluripotent Stem Cells

**DOI:** 10.3390/cells13060534

**Published:** 2024-03-18

**Authors:** Sakib Mohammad, Arpan Roy, Andreas Karatzas, Sydney L. Sarver, Iraklis Anagnostopoulos, Farhan Chowdhury

**Affiliations:** 1 School of Electrical, Computer, and Biomedical Engineering, Southern Illinois University Carbondale, Carbondale, IL 62901, USA; sakib.mohammad@siu.edu (S.M.); andreas.karatzas@siu.edu (A.K.); iraklis.anagno@siu.edu (I.A.); 2 School of Mechanical, Aerospace, and Materials Engineering, Southern Illinois University Carbondale, Carbondale, IL 62901, USA; arpan.roy@siu.edu (A.R.); sydney.sarver@siu.edu (S.L.S.)

**Keywords:** embryonic stem cells, embryoid bodies, mesoderm, deep learning, cell and nuclear morphologies

## Abstract

Pluripotent stem cells can be differentiated into all three germ-layers including ecto-, endo-, and mesoderm in vitro. However, the early identification and rapid characterization of each germ-layer in response to chemical and physical induction of differentiation is limited. This is a long-standing issue for rapid and high-throughput screening to determine lineage specification efficiency. Here, we present deep learning (DL) methodologies for predicting and classifying early mesoderm cells differentiated from embryoid bodies (EBs) based on cellular and nuclear morphologies. Using a transgenic murine embryonic stem cell (mESC) line, namely OGTR1, we validated the upregulation of mesodermal genes (*Brachyury* (*T*): DsRed) in cells derived from EBs for the deep learning model training. Cells were classified into mesodermal and non-mesodermal (representing endo- and ectoderm) classes using a convolutional neural network (CNN) model called InceptionV3 which achieved a very high classification accuracy of 97% for phase images and 90% for nuclei images. In addition, we also performed image segmentation using an Attention U-Net CNN and obtained a mean intersection over union of 61% and 69% for phase-contrast and nuclear images, respectively. This work highlights the potential of integrating cell culture, imaging technologies, and deep learning methodologies in identifying lineage specification, thus contributing to the advancements in regenerative medicine. Collectively, our trained deep learning models can predict the mesoderm cells with high accuracy based on cellular and nuclear morphologies.

## 1. Introduction

The potential of ESCs to differentiate into three primary germ-layers—ectoderm, endoderm, and mesoderm in response to various small molecules or physical cues presents an outstanding opportunity to understand embryogenesis in vitro and derive an unlimited supply of cells for tissue engineering purposes [1,2]. The current methodologies of rapid identification and characterization of cell differentiation into germ-layers, in response to chemical and physical cues, are limited. To identify a particular germ-layer, gene transcriptions or protein expressions are typically evaluated using standard techniques such as qPCR, Western blot, immunofluorescence, etc. However, these validations are time consuming and labor-intensive processes. Additionally, transgenic reporter cell lines expressing fluorescent tags representing endogenous gene activities can also be leveraged to understand lineage commitment. Nevertheless, molecular tag integration requires user expertise and access to specialized facilities. Although the techniques described above are definitive, they may not provide rapid and high throughput analysis and more importantly may not represent a single-cell outcome, rather an ensemble average outcome. It is now well accepted that gene expression patterns, related to specific cell lineage, are also reflected in cell morphology [3]. For example, a cell that belongs to the mesoderm lineage may appear different from ectoderm and endoderm lineages. In addition to cell morphology, as displayed by phase-contrast images, nuclear morphology can also be a unique biomarker for cell classification [4,5]. With the recent advancement, deep learning (DL), a subset of machine learning (ML), has emerged as a powerful tool for image analysis tasks [6]. Within the classes of DL models, convolutional neural networks (CNNs) have demonstrated notable efficiency in such computer vision tasks. CNNs can extract intricate feature information within the pixel data, making them an optimal choice for the detection and classification of the germ-layers from their phase-contrast and nuclei images.

In this study, we derived EBs from OGTR1 mouse embryonic stem cells (mESCs) [7] using the hanging drop culture method [8]. OGRT1 cells are a transgenic reporter cell line that indicate the endogenous activity of *Oct3/4* transcription factor tagged with a green fluorescent protein (GFP) (*Oct3/4* activity indicates self-renewal) and simultaneously indicating endogenous activity of *Brachyury* (*T*) tagged with a red fluorescent protein (DsRed) (*T* expression indicates mesoderm lineage commitment). We used the *T* gene expression to identify mesoderm commitment and appropriately label the phase and nuclear images for image segmentation and classification. Segmentation and classification are addressed by two separate classes of CNNs [9]. Using several CNN models, including Attention U-Net [10] with a DenseNet121 [11] backbone (for segmentation) and InceptionV3 (for classification) [12], we successfully segmented and classified the mesoderm cells and distinguished them from the non-mesoderm (endoderm and ectoderm) cells. Although there are a handful of stem cell morphology prediction studies using DL [13,14,15], to our knowledge, this is the first work with single cells that uses DL methods to capitalize on the cellular and nuclear morphological features in pixel space and identify the germ-layers with high accuracy. The use of DL makes the process accurate, fast, high throughput, and label free.

## 2. Materials and Methods

### 2.1. Cell Culture and EB Formation

A transgenic mESC line, namely OGTR1, that expresses GFP under the promoter of *Oct3/4* and expresses DsRed under the promoter of *T* was used in our study [7]. This cell line was cultured in complete ESC culture medium composed of high glucose Dulbecco’s modified Eagle medium (DMEM) (Thermo Fisher Scientific, Waltham, MA, USA; cat.# 11960069) with additional supplementation of 15% ESC qualified fetal bovine serum (Thermo Fisher Scientific; cat.# 16141-079), 0.1 mM MEM non-essential amino acid (Thermo Fisher Scientific; cat.# 11140050), 2 mM Glutamax (Thermo Fisher Scientific; cat.# 35050061), 1 mM Sodium Pyruvate (Thermo Fisher Scientific; cat.# 11360070), 0.1 mM β-marcaptoethanol (Sigma Aldrich, St. Louis, MO, USA; cat.# M3148), 1% Penicillin Streptomycin (Thermo Fisher Scientific; cat.# 15140122), and finally, 1000 U/mL Leukemia inhibitory factor (LIF) (ESGRO^®^; Millipore, Burlington, MA, USA; cat.# ESG1107). The cells were cultured in an incubator kept at 37 °C with 5% CO_2_. Cells were passaged every 2–3 days at a ratio of 1:20.

EBs were formed with hanging drop culture assay. For setting up the EB culture, each drop contained 600 cells in the absence of LIF (-LIF). This allowed them to aggregate into spherical clusters and differentiate into three germ-layers. After 4 days, the EBs were collected and transferred to 60 mm Petri dishes (VWR, Radnor, PA, USA, cat. # 25384-164) coated with an anti-adherence solution (Stem Cell Technologies, Vancouver, BC, Canada; cat. # 07010). The EBs were grown in -LIF medium for an additional 2 days in suspension and subsequently transferred to gelatin- (Sigma Aldrich; cat. # G1890-100G) coated 6-well tissue culture plates (Thermo Fisher Scientific; cat. # 140675). Differentiated cells were split into single cells and plated on fibronectin- (Sigma, cat. # F2006-1MG) coated glass bottom dishes (Cellvis, Mountain View, CA, USA; cat. # D35-14-0-N) for imaging on day 10. The next day, they were fixed with 4% paraformaldehyde (PFA) (Electron Microscopy Sciences, Hatfield, PA, USA; cat. # 15710), permeabilized with Triton X-100 (Sigma Aldrich, St. Louis, MO, USA; cat. # 93443), and stained with DAPI (4′,6-diamidino-2-phenylindole; Millipore Sigma, cat. #D9542-1MG). Lastly, the fixed and stained cells were mounted with Diamond antifade solution (Thermo Fisher Scientific, cat. # 15810083). The cell culture procedures are summarized in Supplementary Figure S1.

### 2.2. Image Acquisition

**Figure 1:**
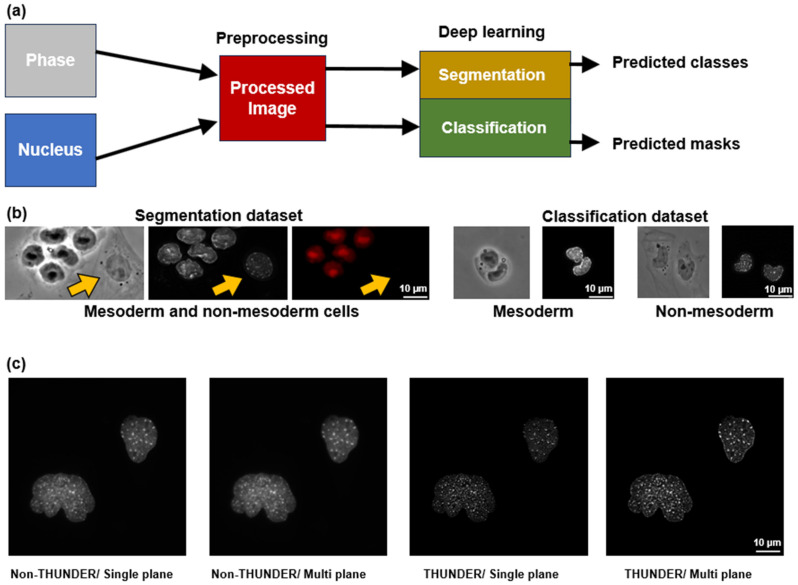
Workflow and a sample of the dataset is presented here. (**a**) Our work is divided into two parts, segmentation and classification of mesoderm and non-mesoderm cells using phase-contrast and DAPI images. (**b**) A sample of the dataset is presented. On the left, phase-contrast, nuclei, and *T* labels are used in the segmentation algorithm. Arrows in yellow indicate a non-mesoderm cell. On the right, we show the mesoderm and non-mesoderm labeled cells used in the classification task. (**c**) A comparison among the non-THUNDER/single plane, non-THUNDER/multi plane, THUNDER/single plane, and THUNDER/multi plane images is shown. The THUNDER/multi plane image captured the complete nuclei content compared to the non-THUNDER single plane image.

Images were acquired with a Leica DMi8 THUNDER Imager epifluorescence microscope with a K8 CMOS camera module and a 40× air objective. We captured phase-contrast images and nuclei images with a DAPI filter and validated the mesoderm positive cells with a Texas Red filter. The image resolution was 2048 × 2048 with a 16-bit depth. The phase-contrast images and the *T* positive images were acquired as single plane images. However, the nuclei images were acquired as a z-stack image set. Our dataset had a total of 25 phase and nuclei images each, and out of those, there were ~165 mesoderm positive cells. It is to be noted here that not every cell expressed *T,* which is evident in Figure 1.

### 2.3. Image Processing

After acquiring the images, the DAPI images were processed using small volume computational clearing (THUNDER-SVCC) on Leica’s in-house software LAS X. THUNDERing the DAPI images allowed us to reject out-of-plane light and acquire unique nuclear morphological features which were subsequently flattened using maximum projection on a single plane. We removed any background noise from mesoderm-positive (*T* labels) images using the sliding paraboloid method in ImageJ Fiji [16,17] so that we had an accurate labeling of which cells were truly mesoderm.

### 2.4. Segmentation Methodology

Images were in 2048 × 2048 resolution and in grayscale format. The high dimensionality of our dataset would have largely increase the computational requirements of the DL models. We could have opted to resize those images to a much smaller resolution (i.e., 256 × 256). But, by downscaling to such an extent, we risked losing a substantial portion of information in our image data. So, we split each of our images into 64 patches with each patch having a resolution of 256 × 256 with no overlapping patches.

#### 2.4.1. Image Processing for Segmentation

During segmentation, the segmentation masks were created using the Labkit [18] plugin in ImageJ Fiji. We created binary masks for both phase and nuclei where 0 indicated non-mesoderm cells and 1 indicated mesoderm cells. Masks were created for both the phase and nuclei separately. We saved the images and masks as *.tiff stacks which ensured every image and its corresponding mask would load in the correct sequence during training. With 64 patches in each image of the 25 phase-contrast and 25 DAPI images, we had a total of 1600 + 1600 = 3200 images as well as 3200 masks. In these images, there were ~165 mesoderm and ~145 non-mesoderm cells. Finally, we normalized the pixel values of the images between 0 and 1 for easy manipulation by the DL models. We normalized the images just before the training loop instead of during loading to prevent data leaking. Supplementary Table S1 shows the image distribution for the segmentation task.

#### 2.4.2. Segmentation Models

We selected U-Net [19] and Attention U-Net as our DL segmentation models since U-Net and its variants have constantly outperformed other CNNs for the task of image segmentation [20].

Since we worked with a modest image data set, we used the ImageNet [21] weights and fine-tuned them for several epochs. In the encoder path of the U-Net models, we used DenseNet121 as a backbone CNN that can utilize the aforementioned weights. We selected DenseNet121 as it outperformed other CNNs such as VGG16 [22] and ResNet50 [23] during our initial attempts.

#### 2.4.3. Segmentation Metrics

For segmentation, we used mean intersection over union (IoU) as our main metric along with the F1 score [24,25]. IoU is an analogous performance metric to accuracy for machine learning tasks. Although accuracy is a decent performance metric for image classification, it cannot capture the extent of correctness in image segmentation. Hence, IoU, in combination with the F1 score, was used to evaluate the performance of the segmentation models in our experiment.

#### 2.4.4. Loss Function for Segmentation

For the loss function, we used the Focal Tversky loss function [26]. It combines the concepts of the Tversky Index [27] and Focal loss [28] for training DL models in tasks such as image segmentation.

The *Tversky Index* is defined as

$$Tversky\;Index=\frac{TP}{TP+\alpha \times FP+\beta \times FN}$$

where *TP* is the number of true-positive pixels (intersection), *FP* is the number of false-positive pixels, *FN* is the number of false-negative pixels, and *α* and *β* are weight factors. 

Focal loss is a modification of the standard cross-entropy loss [29] that assigns higher weights to hard or misclassified examples to focus the training on challenging samples.

The *Focal Tversky loss* combines these two concepts by applying the focal mechanism to the Tversky index. It is expressed as

$$Focal\;Tversky\;Loss={\left(1-Tversky\;Index\right)}^{\gamma }$$

where *γ* is a hyperparameter that controls the degree of focusing or down-weighting.

### 2.5. Classification Methodology

Semantic segmentation models incorporate deeper architectures, thus requiring more computational resources than classification models. However, segmentation is more suitable for our problem in terms of result visualization. Therefore, we selected segmentation as our primary method and classification as the auxiliary method.

#### 2.5.1. Image Processing for Classification

For classification, we cropped the large images to small sizes and saved them to their respective directories (mesoderm and non-mesoderm). The cropped images had both single cells and colonies. Our phase-contrast image dataset had 128 images (65 mesoderm and 63 non-mesoderm), while the nucleus (DAPI) image dataset had 174 images (93 mesoderm and 81 non-mesoderm).

We resized both types of images to 128 × 128 pixels and normalized the pixel values between 0 and 1. The normalization was performed analogously as in the case of segmentation to alleviate numerical issues. To obtain a robust model and to reduce overfitting, we augmented our dataset. We found that augmenting the data twice, once before and later during the training, both randomly and independently, helped the model perform better during testing. Supplementary Tables S2 and S3 present the augmentation parameters before and during training, respectively.

#### 2.5.2. Classification Models

We experimented with several popular CNN models: the VGG16, ResNet50, InceptionV3, DenseNet121, and Xception [30]. However, the VGG16 and ResNet50 did not perform as well as anticipated compared to the other models listed above. So, we selected the InceptionV3, DenseNet121, and Xception models for the classification task. All these CNN models could extract fine grain features from both our phase-contrast and nuclei images and performed well in terms of evaluation metrics.

#### 2.5.3. Evaluation Metrics

In ML, for classification problems, accuracy is the first metric that is generally considered. F1 score, along with accuracy, was also utilized to thoroughly evaluate our models.

### 2.6. Training and Validation of Segmentation Models

We trained our U-Net and Attention U-Net using a DenseNet121 backbone with ImageNet weights. Each of our CNNs was trained for 200 epochs for both phase-contrast and nucleus (DAPI) images. We opted for the Adam [31] optimizer, with a learning rate of 0.0001. We also adopted a 5-fold cross validation method for robust training and validation of our models. The results across all folds were averaged and presented here.

### 2.7. Training and Validation of Classification Models

For classification, the InceptionV3, DenseNet121, and Xception models were utilized. We trained these models with the Adam optimizer with a learning rate of 0.0001 for 100 training epochs. In addition, ImageNet weights were fine-tuned, keeping the first 25% of the layer of each CNN frozen to utilize the already learned low-level features. We used stratified K-fold [32] across all 5 folds so that each fold of the dataset had the same proportions of observations for a particular label. Finally, averages of all the metrics across the folds were taken and presented here.

### 2.8. Explainability of CNN Models

We took a pre-trained InceptionV3 CNN model to show how the images were analyzed during the training and inference stages. The InceptionV3 has a few building blocks called Inception blocks [33]. We took a phase-contrast and a nucleus image and extracted the features from each of these Inception blocks. We also visualized class activation maps of the final convolutional layer of InceptionV3 with the help of Grad-CAM [34]. For these tasks, we utilized ImageNet weights.

### 2.9. Systems Used for Training and Validation

Our segmentation and classification were run on two different systems. The segmentation training was run on a local device with an Intel i7 8700K CPU, 16 GB of system memory, and an NVIDIA GTX 1070ti GPU with 8 GB of video memory. The classification ran on Google Colaboratory with a Pro+ subscription with an NVIDIA Telsa T4 GPU with 16 GB of video memory and 52 GB of system memory. Our codes were written in Python 3’s TensorFlow-Keras [35] deep learning framework. In addition, NumPy [36], Matplotlib [37], Tifffile, and Patchify were used to view and process images. Our workflow is summarized in Supplementary Figure S2.

## 3. Results

### 3.1. Acquisition of Biological Data and the Workflow for DL Processes

We performed the segmentation and classification of our dataset independently. The segmentation is more intuitive visually because we do not have to crop images into single cells and colonies, and we can present the entire view field. However, this is computationally expensive. For this reason, we also performed a classification experiment to investigate how they perform relative to each other. Figure 1a shows the workflow of the computational portion of this project. We utilized two imaging modalities here, the phase-contrast and nucleus image (DAPI). However, they were utilized separately, independent of each other to train the DL models. In Figure 1b, we present the sample dataset for both segmentation and classification with mesoderm and non-mesoderm cells. The left panel (Figure 1b) shows examples of the phase-contrast, nucleus (DAPI), and *T* positive/negative cells for the segmentation process. The right panel (Figure 1b) shows examples of mesoderm and non-mesoderm cells and their respective nuclei images for the classification process. The mesoderm-positive cells expressed *T* while the non-mesoderm cells did not. In addition, for nuclei images, instead of taking single plane images, we acquired z-stacks from a conventional widefield epifluorescence microscope and maximally projected all the stacks on a single plane. However, when we acquired the same image using the THUNDER option using Leica’s THUNDER 3D Imager, the nuclei could be visualized with finer details. Acquiring THUNDERED multi plane images of the nuclei ensured the nuclei features/details were better represented. Figure 1c shows image quality and details gradually improving from left to right.

### 3.2. DL-Based Segmentation Process Predicts Mesoderm Cells Based on Phase-Contrast and Nuclei Images

**Figure 2:**
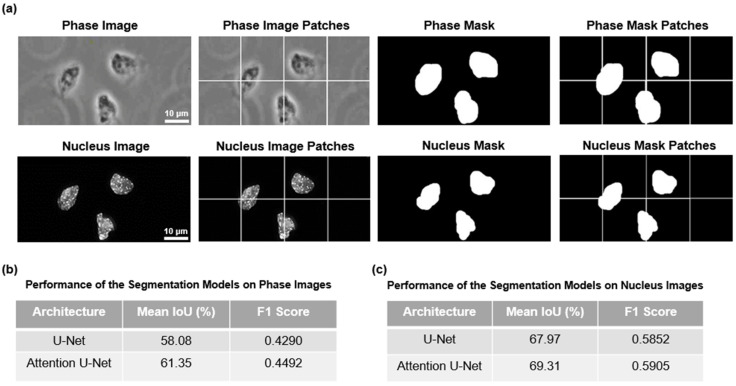
Pre-processing of the dataset for segmentation. (**a**) Patchification of the dataset for the segmentation experiments is shown here. The phase-contrast, the nuclei images, and their respective patches are presented. (**b**) The performance of segmentation models for phase-contrast images is presented here. (**c**) The performance of segmentation models for nuclei images is presented here.

We split the 2048 × 2048 images into 256 × 256 smaller patches so that the DL models could easily handle the images. We did not want to downscale the 2048 × 2048 images to smaller scales such as 256 × 256 because we could lose a substantial portion of the information. Some examples of the image patches for both phase-contrast and nuclei images are presented in Figure 2a along with their respective masks. These patches were subsequently used to train the models.

**Figure 3:**
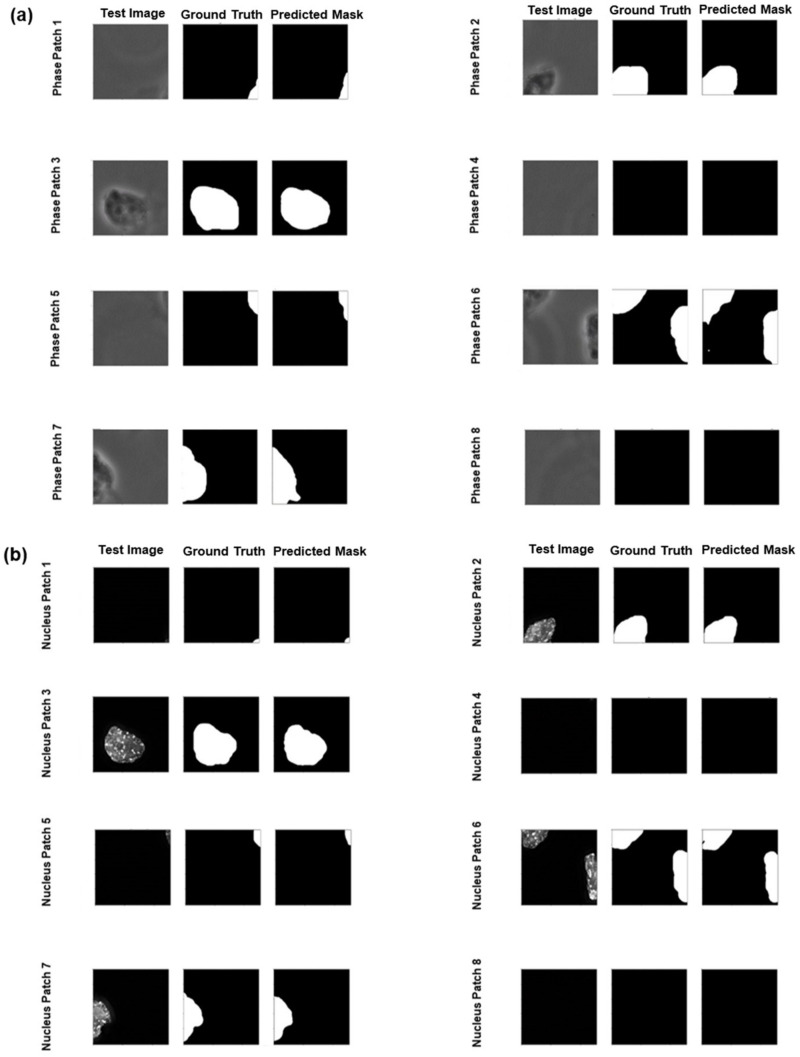
The masks predicted by the Attention U-Net for both phase-contrast and nuclei images are shown. (**a**) The original test images, ground truth masks, and the predicted masks of one of the phase-contrast images are shown. (**b**) The test images, ground truth masks, and the predicted masks of the nuclei images are shown. In some instances, the Attention U-Net predicted the boundary of the nuclei better than the ground truth masks.

Using the pre-processed data as described before, next, we used two CNNs for segmentation purposes. U-Net is a fully convolutional neural network that was developed for segmenting images. In addition to U-Net, we also used Attention U-Net which incorporates a modern attention mechanism [38] found in contemporary transformers [39] that helps to focus better on relevant information. Figure 2b,c show the results of the segmentation of phase-contrast and nuclei images. As expected, the Attention U-Net outperformed the standard U-Net when considering the mean IoU and F1 score. The Attention U-Net focuses on the key features of the phase-contrast and nuclei images and excludes background noises using the attention mechanism. Our models performed well with the nuclei images but struggled with phase-contrast images. Perhaps, this is because the peripheries of the cells in the phase-contrast mode were not well defined, unlike the nuclei images. So, when defining borders around cells in phase-contrast images, the models could not mimic the ground truths as accurately as possible, thus affecting the performance metrics. This is also evident in Figure 3a,b, where we presented the test images for model prediction, the corresponding ground truth masks, and the masks predicted by the Attention U-Net. We see that our model not only accurately predicts where the mesoderm-positive cells were located but also predicts where they were not present. However, the model struggled to define a definite boundary in the periphery of the cells. Nevertheless, since our goal is to identify which cell is mesoderm-positive cells, it is reasonable to state that our model achieved the goal.

From Figure 2b,c and Figure 3a,b it is clear that the Attention U-Net performed well in segmenting the nuclei as opposed to the phase-contrast images. We suspect the nuclei images were more distinguishable with respect to their background compared to cell peripheries in the phase-contrast images. The segmented phase-contrast and nuclei masks, predicted by the standard U-Net model, are shown in Supplementary Figure S3 and Supplementary Figure S4, respectively. We note that our segmentation training on a local computer took about 20 h to complete. We adopted a 5-fold cross validation for the training of the models.

### 3.3. Accurate Classification of Mesoderm and Non-Mesoderm Cells Using Phase-Contrast and Nuclei Images

**Figure 4:**
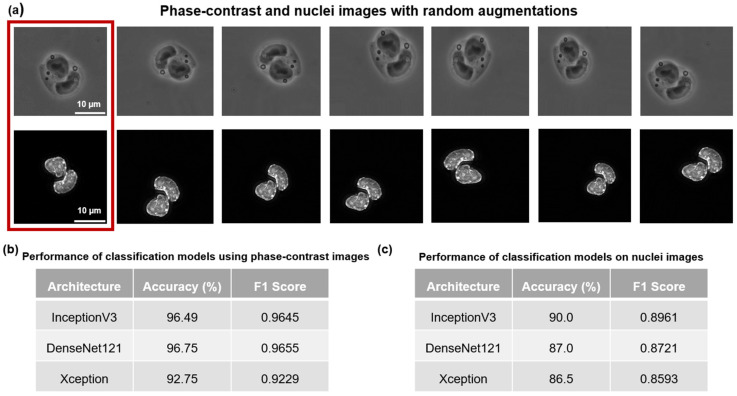
Augmented images of cells in both phase-contrast and nuclei imaging modalities are shown here. The classification task utilized image augmentation. (**a**) The augmentation-free phase-contrast and nucleus images are presented in the red box. All subsequent images were randomly augmented in multiple ways. (**b**) The performance of the three CNN models for phase-contrast image classification is shown here. (**c**) The performance of the three CNN models for nuclei image classification is shown here.

Next, we proceeded to classify the mesoderm vs. non-mesoderm cells. We cropped and saved the phase-contrast and nuclei images of the cells in their respective directories. Using both phase-contrast and nuclei images, we trained the classification models independently from segmentation. In addition, we adopted image augmentation twice in our classification, once before training and again during training to enhance the generalization of our models. The CNN models for classification used in our study include InceptionV3, DenseNet121, and Xception. A sample of the augmented data is presented in Figure 4a and the classification results for different CNN models are presented in Figure 4b,c.

As shown in Figure 4b,c, the InceptionV3 model performed best (90%) for handling nuclei images, while DenseNet121 performed the best (~97%) for the phase-contrast images. Note that the InceptionV3 had very similar performance metrics to DenseNet121 for the phase-contrast images as well. Although the Xception model lagged behind other models in terms of performance metrics, it showed reliable performance across both image modalities. We observe that the classification models were more successful in analyzing the phase-contrast images than the nuclei images. A side-by-side comparison of our segmentation and classification results is presented in Supplementary Figure S5. It is to be noted that we performed the classification task on Google Colaboratory with 5-fold stratified cross validation. Each model took approximately 10 min to train.

### 3.4. Visualization of Extracted Features by DL Model Allows Prediction of Mesoderm Cells

**Figure 5:**
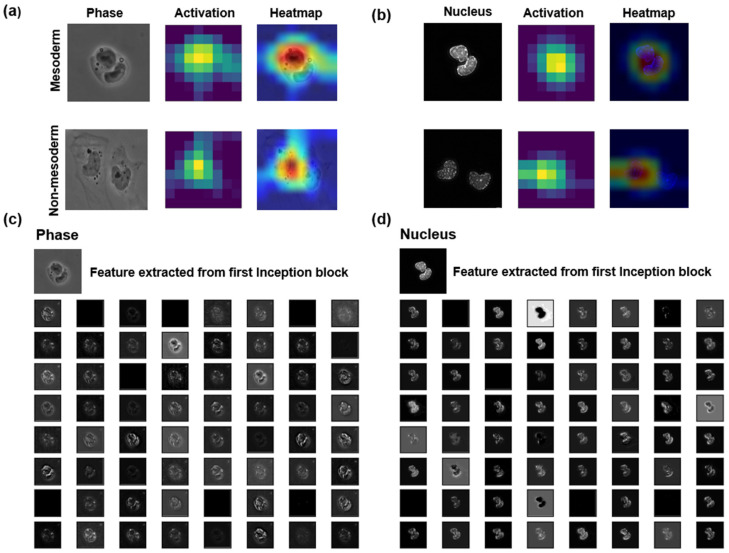
Showing filter activations and extracted features for both phase-contrast and nuclei images using the InceptionV3 model. (**a**,**b**) The activation filter from the final convolution layer of the InceptionV3 shows the decision region for mesoderm and non-mesoderm phase-images and nuclei images, respectively. The activation information is superimposed on the actual images to indicate where the InceptionV3 is focusing. The red, yellow, and blue color maps refer to areas of most, moderate and least importance, respectively, in the heatmap column. The Grad-CAM algorithm aided this visualization. (**c**,**d**) The features extracted by the filters from one of the convolutional layers of the first Inception block of InceptionV3 for both phase-contrast and nuclei images are shown. We observe many distinct characteristics, as shown in coarse grain features. Subsequent blocks have more fine-grained features for both imaging modalities, not presented here.

Generally, the operations within layers in DL models are not clearly visible to the users. We attempted to extract some quantifiable features from deep layers of a DL model. Here, we provide the inner mechanisms that are not readily visible or realized. In Figure 5a,b, we present the class activation maps of the final convolution layer of the InceptionV3 model for both phase-contrast and nuclei images of mesoderm and non-mesoderm cells utilizing the Grad-CAM method. The heatmaps show the regions of interest that the DL model identifies as important for making a class prediction (mesoderm or non-mesoderm). It is evident that the activation is different for all four images. Therefore, it is acceptable to say that the same model will process different images differently while making a prediction. We can infer that these areas on the respective images have the characteristics that define mesodermal vs. non-mesodermal lineages. Figure 5c,d shows the features (or the details of the characteristics) extracted from one of the convolutional layers of the first Inception block of the InceptionV3 model for the phase-contrast and nuclei images, respectively. With an identical architecture with different inputs, the extracted features by the InceptionV3 model varied a lot, although the detailed mechanism of feature extraction remained elusive. The outputs of the rest of the blocks of the InceptionV3 for the phase-contrast and nuclei images are presented in Supplementary Figure S6 and Supplementary Figure S7, respectively.

## 4. Discussion

The primary objective of this project is to accurately identify cells transitioning along the mesodermal lineage. Although our segmentation results were not high enough (particularly the F1 score), our robust classification results allowed us to meet our primary objective. In Figure 3, we compared the masks generated by our models with the ground truth models. Our model may not precisely mirror the shape of the ground truth masks because they were hand-drawn and had less-than-perfect overlap around the boundaries. In addition, the segmentation task already required high precision from our models, and the segmentation process being as stringent as it is, it penalized even minor misclassification of pixels, thus further affecting our performance metrics. To mitigate this issue, future work will employ fully automated mask constructions. Despite these challenges, it is crucial to recognize that the primary objective of this project is to accurately identify cells transitioning along the mesodermal lineage. The focus is not on the precise formation of the mask around each cell but rather on the reliable detection of mesoderm cells themselves.

For the segmentation task, in addition to U-Net and Attention U-Net, we utilized U-Net++ [40]. However, we could not proceed with this model because of memory constraints. In addition, we employed the VGG16 and ResNet50 as backbones for the U-Net and its variants in addition to DenseNet121. Nevertheless, neither of the models yielded favorable results.

During the training of both the U-Net and Attention U-Net models, we implemented both shallow tuning and deep tuning [41] of ImageNet weights. For our experiments, we proceeded with the deep tuning method because of its superior performance compared to shallow tuning of the weights, where only the final layers of the encoder model are trained as opposed to the entire encoder model. This was expected because our dataset is fundamentally different than the ImageNet dataset on which the original weights were trained. We initialized our experiments with the ImageNet weights as the starting point of deep tuning and trained them for an additional 200 epochs, which resulted in excellent model performance. Moreover, we also used random weights initialization, and after comparing the results with the deep tuning method, we decided to go forward with the latter for better performance. We believe having a small dataset was not sufficient to tune the weights if they were randomly initialized. The number of epochs along with all the hyperparameters was decided iteratively to reduce underfitting (a phenomenon where the model underperforms on both training and testing data) and overfitting (a phenomenon where the model performs well on training but not on testing data). It would have been ideal if we used image augmentation techniques to improve the generalization of our models. However, due to memory constraints, we could not pursue that direction. We believe loading and processing a high number of high-definition 16-bit images and their corresponding masks and performing a 5-fold cross validation led to such memory constraints.

Segmentation and classification are two distinct training approaches that should not be compared in terms of performance metrics alone. Both of these approaches successfully identified the cells that are committing towards mesoderm lineage. The segmentation, although it takes a lot of time to compile, is visually more intuitive in the sense that we are actually seeing which cells are mesoderm from the full-view field of a large image. In contrast, the classification is not visually intuitive like the segmentation because we are essentially cropping from the full-view field of a large image and then feeding this into a model for training. Because of this, the training becomes considerably fast. Both methods are reliable, but the choice between them ultimately depends on the user’s preference for visual articulation and available computational resources. We observe an opposing trend when considering phase-contrast and nuclei images during segmentation and classification. During segmentation, the performance metrics for the nuclei images were higher than the phase-contrast images. In contrast, during classification, the phase-contrast images exhibited higher performance metrics than the nuclei images. Perhaps, the nuclei (DAPI) images worked better for segmentation because their boundaries were in high contrast when compared to their background. On the other hand, phase-contrast images for classification had richer morphological features/patterns than nuclei (DAPI) images and thus provided more context for classification.

We also explained the inner workings of the neural net models while analyzing the images. We provided a few activation kernels and heatmaps indicating where the model is focusing on the image while making a class decision. In our future works, we intend to emphasize more on presenting the mechanisms of the DL models while making such class decisions.

One caveat of our study is that we have only used a single mESC line to train our model. In the future, we plan to use several mESC lines, including primary mESCs, to train and validate our model. Furthermore, we will employ similar approaches to detect all three germ-layers, including ectoderm and endoderm, to predict early lineage commitment with high accuracy, thus contributing to rapid and high-throughput early lineage commitment prediction of pluripotent cells in response to chemical and physical cues. Finally, this project’s real-life implications can be found in the field of regenerative medicine. Our methods can be utilized for high-throughput drug screening that influences stem cell differentiation. Although we did not study any human pluripotent cell line, similar approaches, as demonstrated here, can be further adopted in personalized medicine for early lineage prediction of patient-specific induced pluripotent stem cells. The methods discussed here can significantly improve the efficacy of these procedures.

## 5. Conclusions

Taken together, we have demonstrated how to leverage deep learning models to reliably predict mesoderm lineage that arises from mESCs. We employed both segmentation and classification processes independently to identify differentiated early mesoderm cells from a pool of heterogeneous cell populations with high accuracy.

## Data Availability

The data are available from the corresponding author upon request.

## Supplementary Materials

The following supporting information can be downloaded at: https://www.mdpi.com/article/10.3390/cells13060534/s1, Figure S1: Cell culture workflow, detailing the steps from culturing ESC to immunostaining. Figure S2: Our laboratory and computation-based workflow. Figure S3: The phase-contrast images, ground truth masks, and predicted masks by the segmentation model U-Net are shown here. Figure S4: The nuclei images, ground truth masks, and predicted masks by the segmentation model U-Net are presented here. Figure S5: Performance comparison of the CNN models. Figure S6: Features extracted from Inception blocks 2–8 of the InceptionV3 CNN model are visualized for a phase-contrast image. Figure S7: Features extracted from Inception blocks 2–8 of the InceptionV3 CNN model are visualized for a nucleus image. Table S1: Image distribution for segmentation. Table S2: Augmentation parameters before training. Table S3: Augmentation parameters during training.
