# Supplementary material for: Deep Learning Powered Identification of Differentiated Early Mesoderm Cells from Pluripotent Stem Cells
Source: Cells. 2024 Mar 18;13(6):534. doi: 10.3390/cells13060534 (PMC10969030; doi:10.3390/cells13060534)
Supplement: Supplementary file 1 [file cells-13-00534-s001.zip › cells-2914548-supplementary.pdf]

Supplementary Materials

for

Deep Learning Powered Identification of Differentiated Early Mesoderm cells from Pluripotent Stem Cells

Sakib Mohammad, Arpan, Roy, Andreas Karatzas, Sydney L. Sarver, Iraklis Anagnostopoulos, Farhan Chowdhury*

*Correspondence: farhan.chowdhury@siu.edu

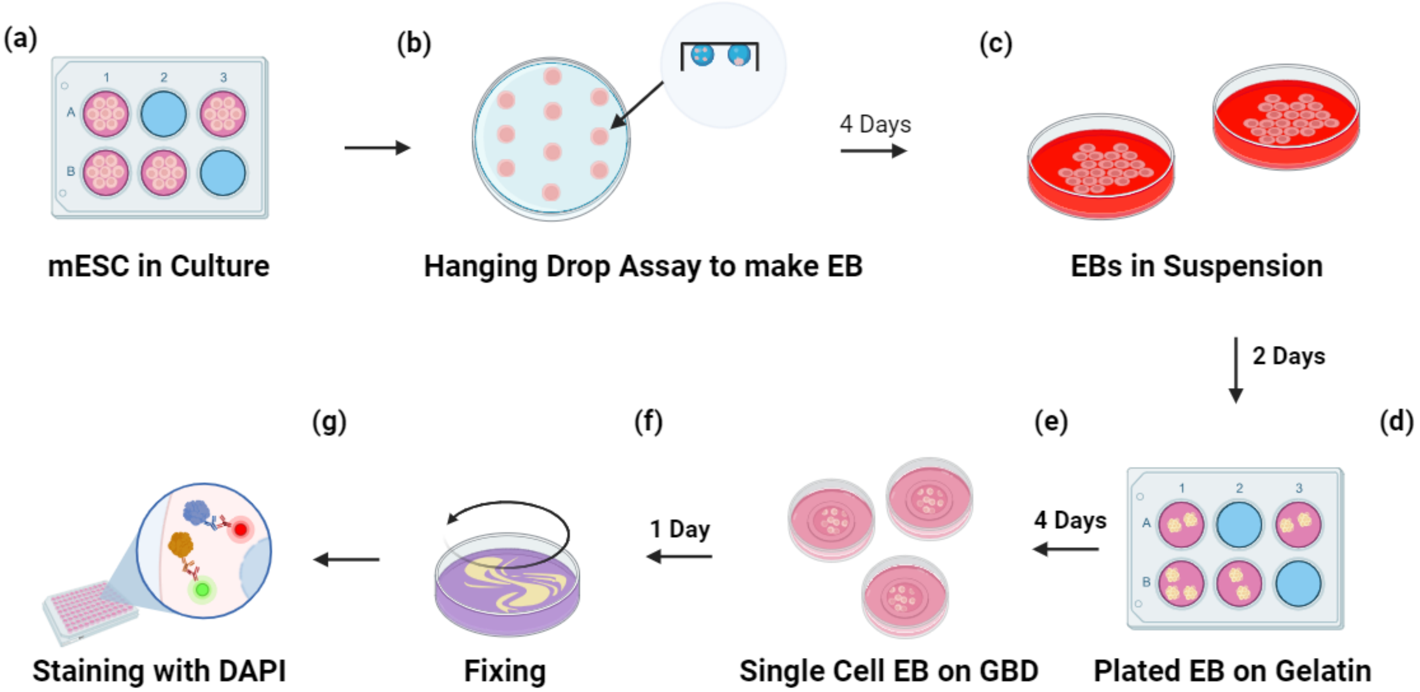

Figure S1. Cell culture workflow, detailing the steps from culturing ESC to immunostaining. (a) mESC culture in 6-well plate. (b) Hanging drop assay to make EB from ESC. (c) EBs cultured in suspension allowing them to grow. (d) Culturing EBs in 6-well plate coated with gelatin. (e) Single cell EBs on glass-bottom dishes to initiate fixing and staining procedures. (f) Fixing EBs with PFA. (g) Immunostaining with DAPI.

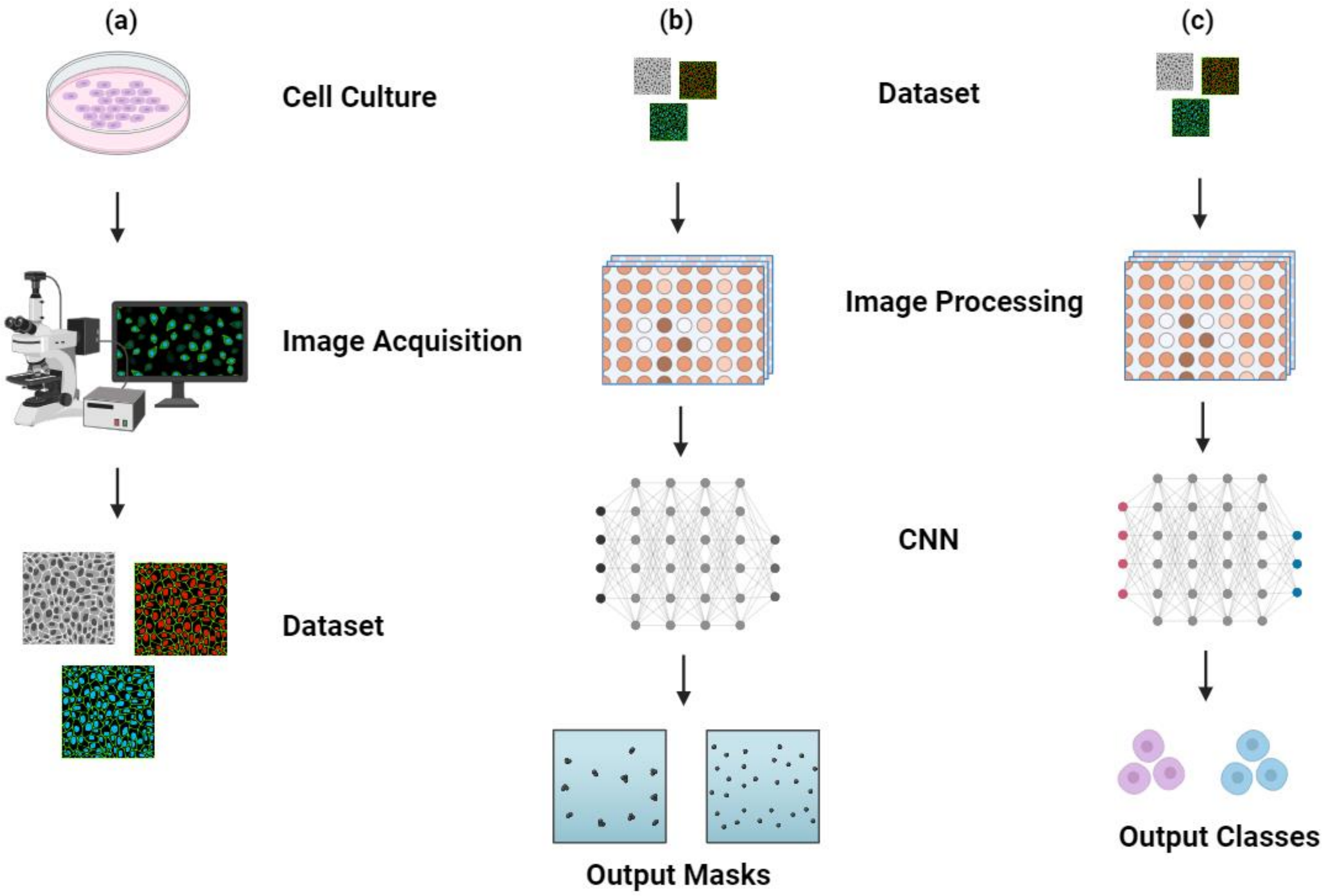

Figure S2. Our laboratory and computation-based workflow. (a) Cell culture related and image acquisition. (b) Segmentation workflow (c) Classification workflow.

U-Net Phase Patches

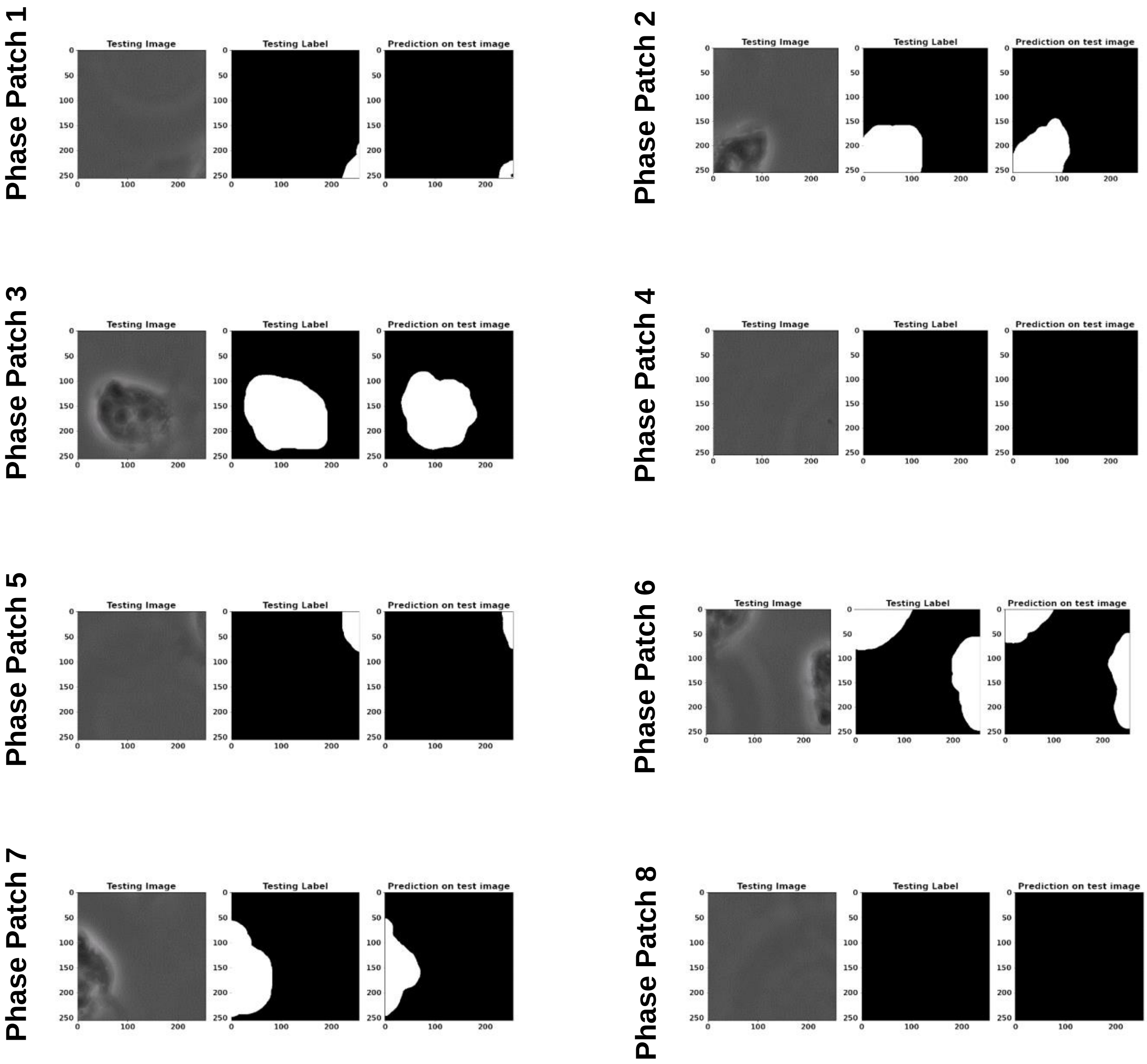

Figure S3. The phase-contrast images, ground truth masks, and predicted masks by the segmentation model U-Net are shown here.

U-Net Nuclei Patches

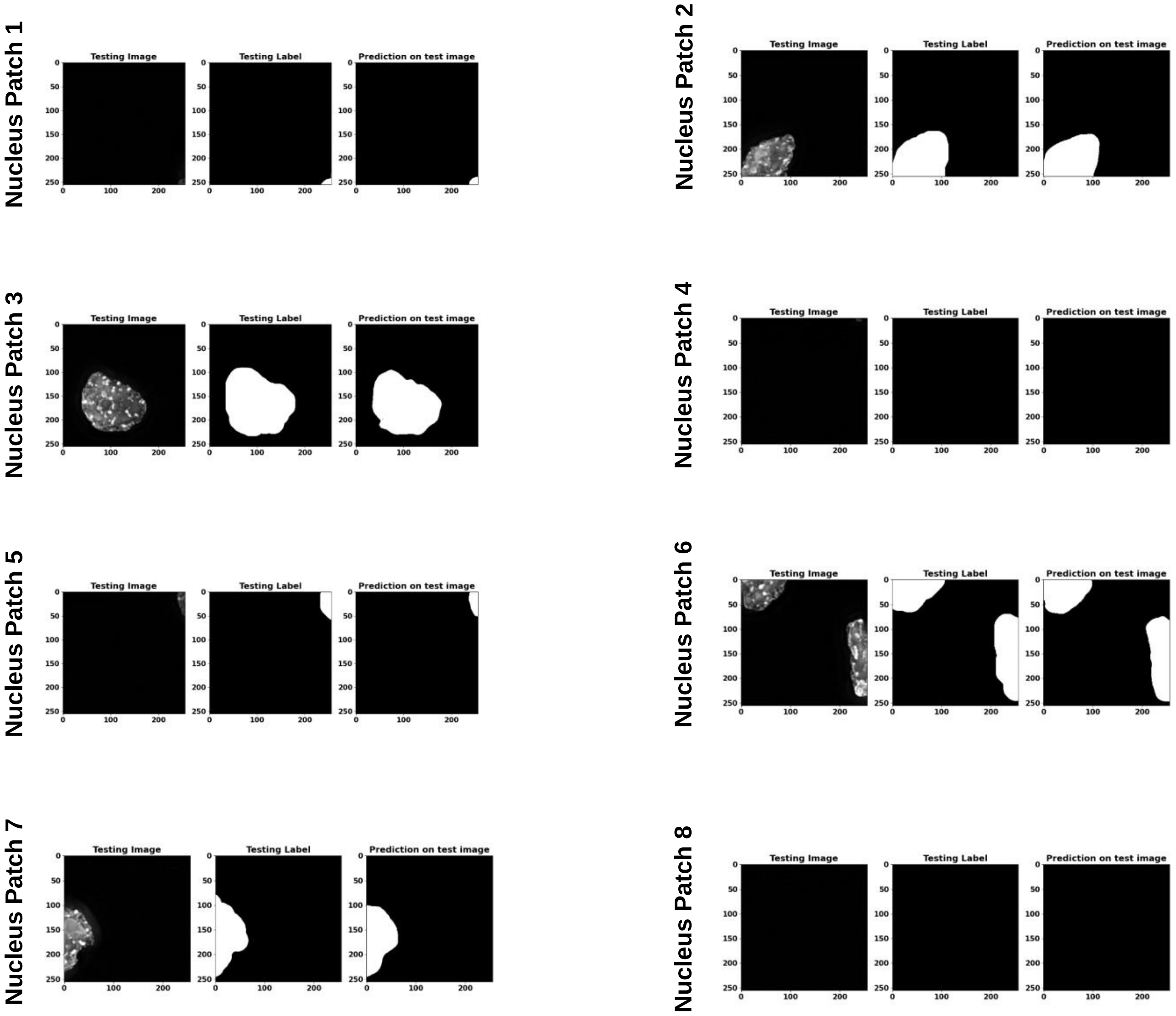

Figure S4. The nuclei images, ground truth masks, and predicted masks by the segmentation model U-Net are presented here.

(a)

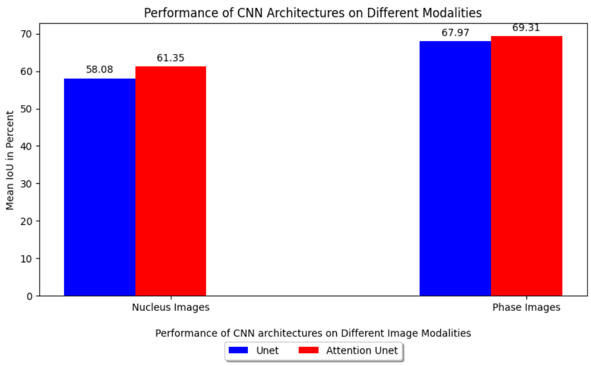

(b)

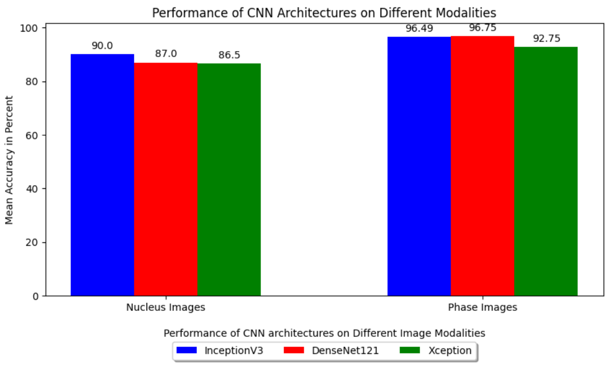

Figure S5. Performance comparison of the CNN models. (a) Mean IoU comparison for nuclei and phase images between U-Net and Attention U-Net segmentation models. (b) Accuracy comparison for nuclei and phase images among InceptionNetV3, DenseNet121 and Xception classification models.

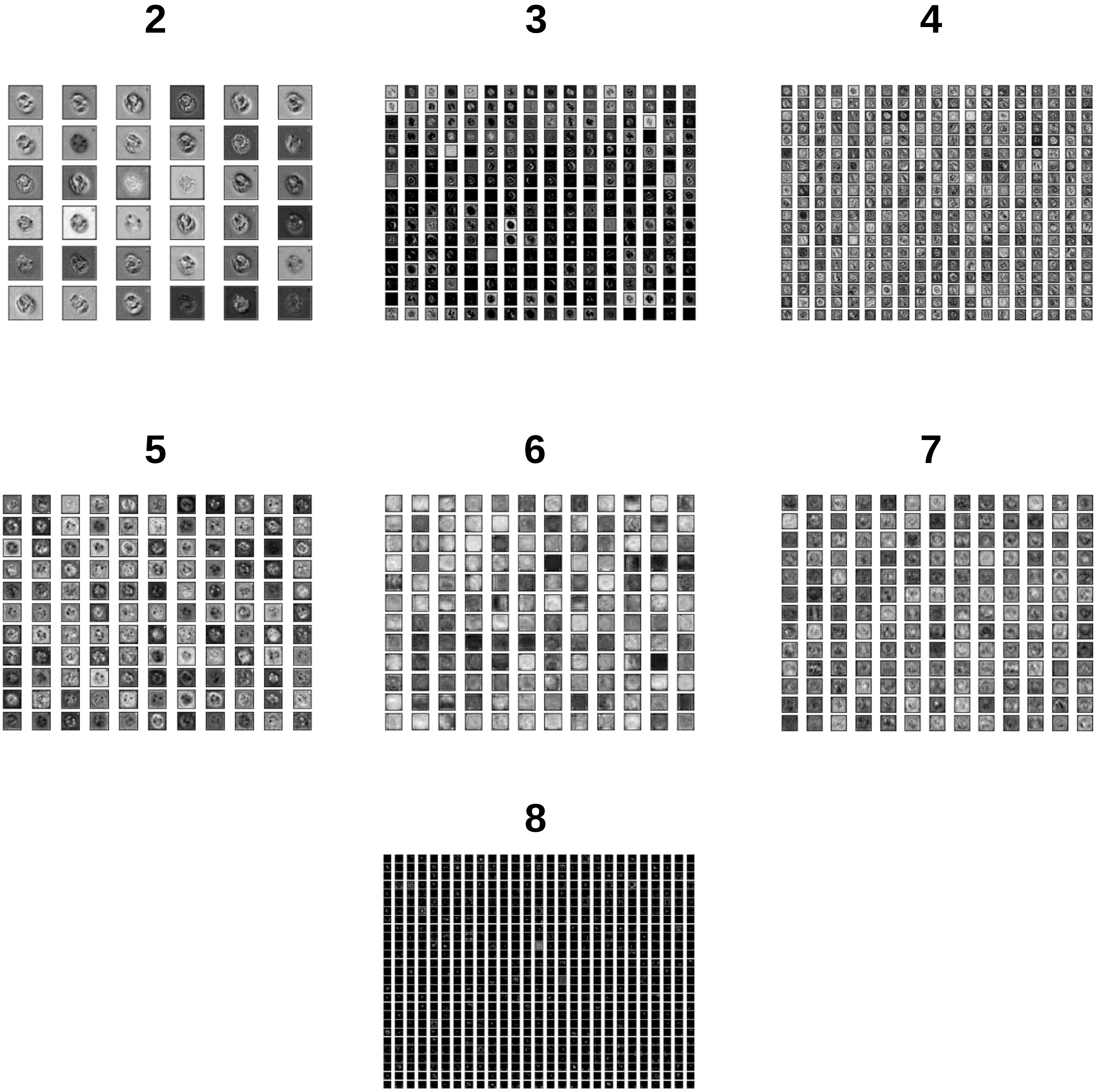

Figure S6. Features extracted from the Inception blocks 2-8 of InceptionV3 CNN model are visualized for a phase-contrast image.

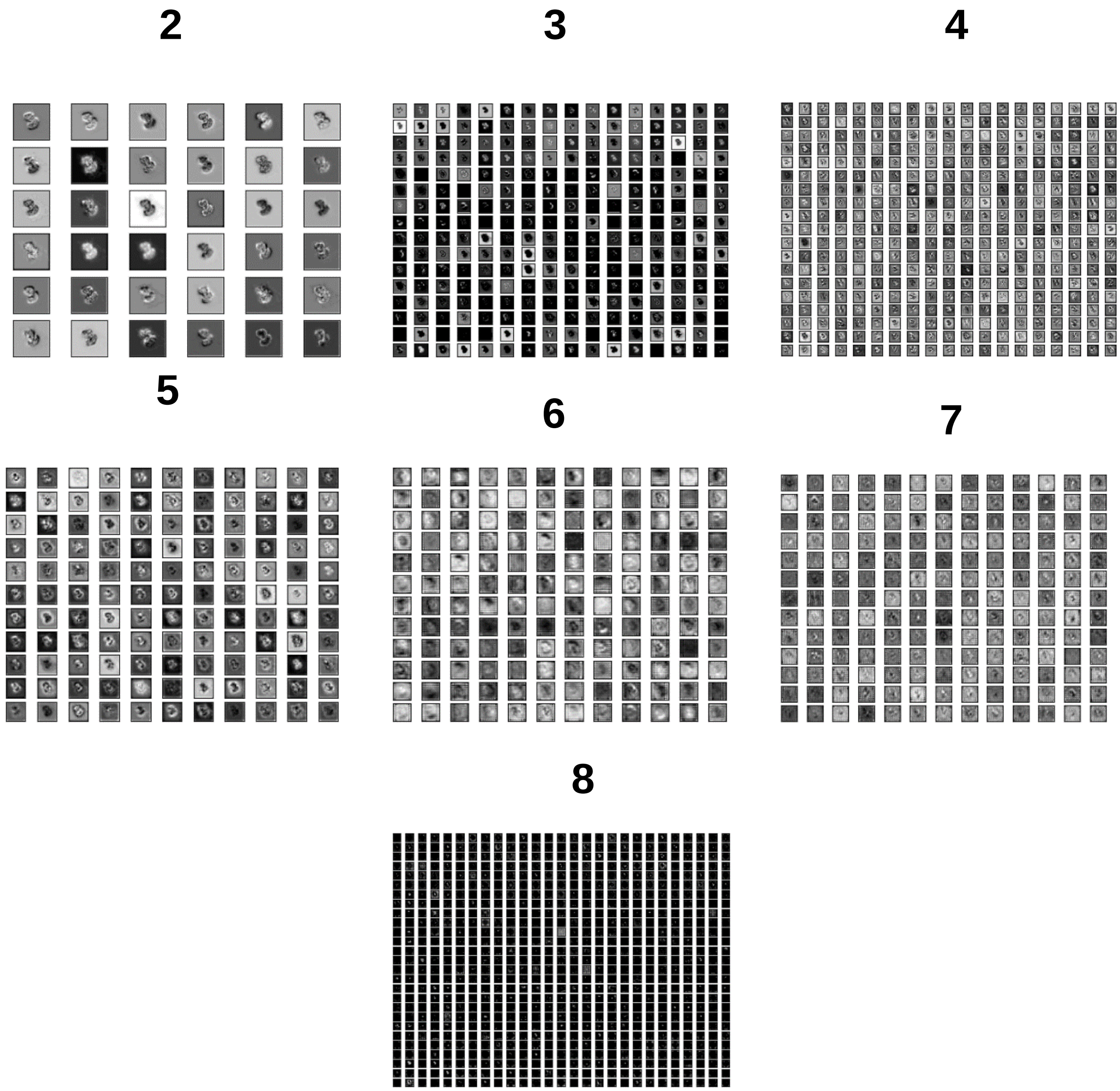

Figure S7. Features extracted from the Inception blocks 2-8 of InceptionV3 CNN model are visualized for a nucleus image.

Table S1. Image distribution for segmentation.

**Image Type**	**Number**
2048*2048 Phase images	25
2048*2048 Nucleus images	25
2048*2048 Mesoderm images	25
2048*2048 Phase image masks	25
2048*2048 Nucleus image masks	25
256*256 Phase image patches	1600
256*256 Nucleus image patches	1600
256*256 Phase image mask patches	1600
256*256 Nucleus image mask patches	1600

Table S2. Augmentation parameters before training.

Augmentation	Range
Random Rotation	20
Width Shift	0.15
Height Shift	0.2
Random Shear	0.2
Random Zoom	0.15
Horizontal Flip	Yes
Vertical Flip	Yes

Table S3. Augmentation parameters during training.

**Augmentation**	**Range**
Random Rotation	90
Width Shift	0.3
Height Shift	0.3
Random Shear	0.5
Random Zoom	0.3
Horizontal Flip	Yes
Vertical Flip	Yes
